# Difficult Diagnosis

**Published:** 1890-03-22

**Authors:** 


### DIFFICULT DIAGNOSIS.

Dr. J. Mitchell Bruce contributed to the *Practitioner* some time ago a rather lengthy paper on "difficult diagnosis." He remarks that the first cause of difficulty in diagnosis is the novelty and rarity of a disease. New, or at any rate novel diseases are still being discovered, such as myxœdema and actinomycosis, and it may take years before the general body of the profession becomes sufficiently acquainted with their characters to recognize them readily. Ignorance of methods of investigation is a source of failing diagnosis. There are few of us who can claim a practical familiarity with all the instruments and appliances now used in medicine and surgery as aids to diagnosis. Therefore we suppose, although Dr. Mitchell Bruce does not say so, that specialists must flourish to the end. Intrinsic difficulties will occur. However strongly we may suspect the existence of certain diseases, we cannot be positive about them. In obscure cases experience must come into play, although unfortunately even experience is frequently baffled. Temporary difficulties may also arise. Time is here required. As for instance to declare the character of a fever, to reveal the nature of a cancer, or to evidence the presence of a hepatic abscess. Difficulties in connexion with the patient may be experienced. The infant, the child, the aged, the unconscious, the aphonic, the foreigner, all present difficulties! In this connexion Dr. Bruce refers to the manner in which the nervous temperament occasionally obscures diagnosis. Spasms, convulsive cries, relaxation of the sphincters, may in nervous subjects, especially in Orientals, take the place of the shiver or vomit of the more phlegmatic Anglo-Saxon. Uncommon or erratic types of disease often lead to difficult diagnosis. Most maladies present somewhat different symptoms in different people; much being due, as we believe, to temperament and constitution of the sufferers. This is especially seen in febrile complaints and in diphtheria, of which there are mild and severe types. Neglect of details also occasions difficulty. Haste is commonly accountable for this. We need not stay to mention the numerous details which the physician should attend to, many of which are noted by Dr. Bruce. It, indeed, appears right that all the organs should be examined whenever a client presents. How this is to be done by the prescribing out-patient physician, who works against time, Dr. Bruce does not inform us. Magnifying details may also lead to wrong diagnosis. This, again, must be a matter of experience. The author observes: "Many excellent observers lose their heads entirely over little points in physical examination." Subtle causes are referred to under drain-poison and malaria. No doubt drain-poison is responsible for various disorders, such as obscure attacks of sore throat, diarrhœa, and, according to Dr. G. Johnson, renal disease. And malaria has been credited as the cause of every malady for which no other reason can be adduced. "Malaria," says Dr. Bruce,

"is still meeting us at every turn;" but, as the author truly remarks, most of the cases now originate abroad. Among inorganic poisons, the most frequent causes of difficult diagnosis are lead and arsenic. The difficulty which arises in connection with these cases is the discovery of the source of the toxic substance. Lead has been found to contaminate tinned meats and soda water. Arsenical poisoning has been known to arise from wall papers. Obscure diathesis is another source of difficult diagnosis. Gout lies "at the bottom" of a large number of complaints. "It comes before us in the asthmatic child, in the young man or woman complaining only of recurrent headache . . . in the middle-aged man of full habit . . . who is conscious of irregularity and palpitation of the heart . . . in the feeble aged patient." Rheumatism occasionally has peculiar irregular manifestations which are difficult of diagnosis. Sore throat is common at the commencement of acute rheumatism, and orchitis may have a like origin. In this connection the author does not say anything on the subjects of either specific or scorbutic diathesis, which are "at the bottom" of more obscure maladies than gout. Incomplete diagnosis is next mentioned. This, however, might well have come under one of the other heads, as neglect of details, for instance, and haste. Jumping at conclusions is next mentioned, and the author says, "In another and very instructive class of cases, diagnosis is difficult because it is easy." Several cases to the point are mentioned. Pleurisy mistaken for congestion of the liver. Optic failure attributed to specific malady, the case being one of Bright's disease. The conclusion of the paper is headed General Suggestions for Removing Difficulties and Preventing Failure. These resolve themselves into "keeping abreast of medical science," and approaching every clinical problem—and every case is a problem—not with preconceived notions, nor stopping short when we have discovered a few symptoms or one lesion sufficient to account for the condition.

